# Extirpation of the Uterus by Mistake for Ovarian Tumor

**Published:** 1867-10

**Authors:** 


### Miscellaneous.

### Extirpation of the Uterus by Mistake for Ovarian Tumor.

Dr. E. Krakowizer presented to the New York Pathological Society the body of a uterus, in the walls of which was imbedded a large fibroma, taken from a woman forty-eight years of age. She was always in most excellent health, and menstruated regularly up to the time of the operation. About two years ago she felt that her health was becoming somewhat impaired. One year ago a swelling in the lower part of the abdomen appeared; it did not increase, however, to any considerable degree, or with any marked rapidity, and its presence only occasioned her uneasiness.

Dr. K. saw her for the first time about six weeks ago. The tumor, which was about the size of a child's head two years old, was felt occupying the lower part of the abdomen. It was smooth, elastic, painless on pressure, and movable from side to side. On making a vaginal examination, it was found that the mass bore down behind the symphysis pubis, and had crowded the uterus backward. The os was felt on the posterior part of the tumor. A uterine sound entered with great facility to the depth of two inches and a half behind the tumor. When the sound was grasped by one hand and the tumor held with the other, it seemed to move independently; I therefore concluded that I had a simple ovarian tumor to deal with, and that the uterus was normal and unconnected with it. I supposed that the pedicle was a pretty short one. No examination per rectum was made.

The condition of things was explained to the woman, and she most decidedly preferred to have a radical operation undertaken. Dr. K. then called in Dr. Kammerer, who made an examination, and corroborated the former opinion in every respect, except that he thought that the tumor originated from the left side, while Dr. K. was of the opinion that it came from the right side.

After chloroform was administered, an incision was made midway between the umbilicus and symphysis pubis to the extent of four inches, and the tumor was presented. At the previous examinations, never having felt any fluctuation, Dr. K. was of the opinion that the walls of the cyst were very thick. When the peritoneal cavity was opened sufficiently wide so as to introduce the hand, it was found that the omentum was adherent in several spots on the surface of the tumor. On the left side a band was detected, with a somewhat cylindrical yielding mass in it, which seemed to be the dilated fallopian tube of the left side. On bringing the hand between the promontory and the tumor, the mass was found to be connected most intimately with the cervix uteri. Before that was done, however, a trocar was plunged into the mass, and on introducing the trocar, Doctor K. became aware that he had to deal with a solid tumor, as no fluid escaped and the end of the trocar was firmly held fast. A great deal of venous oozing took place from the spot. Between the promontory of the sacrum and posterior aspect of the tumor Dr. K. could feel plainly the left ovary, and he could also ascertain that the mass was a continuation of the cervix uteri. The uterine sound was passed to the depth of two inches and a half; it was also evident that the tumor was springing from the lower portion of the body of the uterus, which a little above the inner os was swelling out rapidly into a globular tumor.

The question then arose whether it was better to desist from the operation or go on. The latter was decided on. Dr. K. ligated both fallopian tubes, and of course part of the broad ligament, and after the ligation of these bands, and when the tumor became more movable, he lifted it out of the abdominal cavity, pulling it well up above the symphysis pubis, so that the neck of the tumor could be seen and felt. He then carried the chain of the écraseur around it, and proceeded very slowly to close it. He was fully three-quarters of an hour in accomplishing this, in the fear that by proceeding more rapidly hemorrhage might ensue. After the chain had worked through the rest of the womb, that is, the cervix being still upon the stretch above the symphysis, no hemorrhage was visible, but as soon as the stump was fairly liberated the whole field of the operation was deluged with blood. The stump was again grasped with the forceps, and both uterine arteries were secured, but the uterine veins, as well as several sinuses in the cervix itself, continued to pour forth blood, and these with great difficulty were at length secured. For greater security against accident, a silver wire was then twisted around the end of the stump, and the ends brought out of the wound.

The loss of blood was considerable, but the pulse did not indicate an anæmic condition of the body.

The operation took fully two hours and a half. The wound was closed with five silver wire sutures. A hypodermic injection of Magendie's solution was then administered which caused an hour's sleep. Four drops of Magendie's solution were given every hour during the night. After midnight she became restless and vomited several times. It was then very evident that peritonitis was extending rapidly. A second hypodermic injection was made, and large doses of Magendie were given. At four o'clock she died. No post-mortem examination was made.

The specimen has been incised by a longitudinal cut, and it will be seen that a large fibroma is imbedded in the walls of the uterus, and that this mass has nothing to do with the cavity itself."—*Medical Record*, Sept. 1, 1867.

